# Clinical and demographic characteristics of participants in a hepatitis C treatment trial in rural Kentucky: How policies around treatment access may impact elimination efforts in the United States

**DOI:** 10.1111/jrh.70128

**Published:** 2026-02-07

**Authors:** Jennifer R. Havens, Brittney D. Williams, Takako Schaninger, Virginia A. Shepherd‐Tackett, Michelle R. Lofwall, Michele Staton, Sharon L. Walsh, Hannah K. Knudsen

**Affiliations:** ^1^ Department of Behavioral Science University of Kentucky College of Medicine Lexington Kentucky USA; ^2^ Center on Drug and Alcohol Research University of Kentucky Lexington Kentucky USA; ^3^ Department of Internal Medicine Division of Infectious Disease University of Kentucky College of Medicine Lexington Kentucky USA

**Keywords:** Appalachia, direct‐acting antivirals, hepatitis C, Medicaid policy

## Abstract

**Introduction:**

The advent of curative direct acting antiviral (DAA) drugs to treat those actively infected with the hepatitis C virus (HCV) has allowed for discussion around HCV elimination. Restrictive state‐by‐state policies for the coverage of DAAs for Medicaid recipients may hamper elimination efforts in the United States by limiting access to these curative treatments.

**Methods:**

The purpose of the current analysis was to examine the sociodemographic, drug use and clinical characteristics of participants in the Kentucky Viral Hepatitis C Treatment (KeY Treat) study in the context of Medicaid policies in the United States. The goal of KeY Treat was to reduce barriers to accessing curative DAAs by providing screening and treatment free of charge.

**Results:**

Results suggest that fewer than one in five KeY Treat participants would be eligible for HCV treatment in states without Medicaid expansion. A third of KeY Treat participants were actively injecting drugs and 40% indicated recent drug use, which would negatively impact their ability to easily access treatment in seven US states. More than 85% of KeY Treat participants started treatment the same day as screening. However, same‐day test and treat models would not be possible in almost half of US states because of preauthorization requirements that limit the ability of providers to employ innovative point‐of‐care RNA screening.

**Conclusions:**

As an elimination plan takes shape in the United States, it is clear that it will be necessary to remove all restrictions for accessing treatment to allow for meaningful increases in HCV treatment uptake and cure.

## INTRODUCTION

Worldwide elimination of the hepatitis C virus (HCV) as a public health threat is possible given the efficacy of curative direct acting antivirals (DAAs) for the treatment of active HCV infections.^1^, ^2^ Current elimination goals set forth by the World Health Organization call for 80% reductions in transmission by 2030, as well as 65% reductions in mortality, with a focus on scaling up access to DAAs and treating those transmitting HCV in order to curb the spread.^3^ The primary mode of transmission in the United States is sharing of equipment used to prepare and inject drugs,^4^, ^5^, ^6^ and a dramatic shift in the epidemic curve for HCV has occurred as a result of the opioid epidemic, with the majority of new infections among young people who inject drugs (PWID).^7^ Further, recent modeling data suggest that incident HCV infections among PWID are current outpacing the number of individuals cured in the United States.^8^

Limited engagement of PWID in curative HCV treatment in the United States may challenge elimination efforts as insurance policies surrounding access to care represent an enormous barrier to treatment seeking.^9^, ^10^ A recent analysis suggests that the majority of PWID in the United States are eligible for insurance through Medicaid,^11^ which is funded jointly by the federal government and the states. This joint funding model means that a state can place restrictions on what services are covered within that state, and many states have enacted numerous restrictions specific to accessing HCV care. According to the Center for Health Law and Policy Innovation,^12^ in 2025 more than a third of all states require a prior authorization before initiating HCV treatment, 12% of states had illicit drug abstinence requirements, three states still require specialist treatment, and a quarter of US states limit retreatment for patients who are reinfected.^12^

While state Medicaid restrictions place an undue strain on PWID attempting to access HCV care, some PWID are simply unable to obtain Medicaid coverage at all. For example, there is an ongoing barrier in 10 states that have not expanded Medicaid under the Affordable Care Act to cover uninsured adults.^13^ Medicaid expansion is critically important for PWID, as an analysis undertaken after implementation of the ACA reported that only 37% of PWID had Medicaid coverage in non‐expansion states compared to 87% of PWID in Medicaid expansion states.^14^ In Kentucky alone, Medicaid expansion led to a 158% increase in coverage among people who use drugs.^15^

The purpose of the current analysis is to examine the baseline demographic, drug use, and clinical characteristics of participants enrolled in an HCV treatment trial in a rural community in Appalachian Kentucky adversely impacted by the opioid epidemic in the context of state‐level policies around accessing curative HCV treatments. Kentucky's state policy context is somewhat mixed in terms of barriers to HCV care. Kentucky is a Medicaid expansion state, which has dramatically increased health insurance coverage for PWID.^15^ However, Kentucky's Medicaid program requires prior authorization for DAAs and has retreatment restrictions.^16^ Understanding the demographic, drug use and clinical characteristics of those engaging in an HCV treatment trial may help guide Medicaid policy around treatment eligibility and provide a blueprint for HCV treatment delivery in resource‐constrained rural areas of the United States.

## STUDY DESCRIPTION

The purpose of the Kentucky Viral Hepatitis C Treatment (KeY Treat) study (NCT03949764) was to provide HCV treatment to all actively infected individuals in Perry County, Kentucky (population 27,133).^17^ Perry County was chosen as the study site because HCV exposure and HCV‐related risk behaviors were well characterized in the community.^18^ Further, Perry County operates a harm reduction program,^19^ which team members determined was integral to preventing reinfection. Perry County has been designated as a nonmetro county, with a rural–urban continuum code of 7 (urban population of 5000–20,000, not adjacent to metro area).^20^ All study‐related medications and care were provided free of charge, thus removing restrictions for accessing curative therapy and a same‐day test and treat model was employed. For the detailed clinical protocol, please see Havens and colleagues.^21^ Briefly, participants were recruited beginning in September 2019 and all study related activities were complete as of June 2025. A variety of passive and active recruitment mechanisms, including participation in previous studies, flyers, advertisements in local media, a newspaper and television segment, and through word of mouth. Study staff also conducted regular HCV screening events in the local syringe services program (SSP) and medication for opioid use disorder (MOUD) clinics. Those eligible for the study were at least 18 years of age and HCV RNA‐positive; pregnant women were excluded.^21^

## STUDY PARTICIPANTS

**FIGURE 1:**
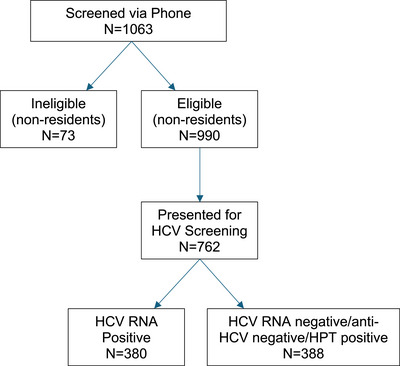
Enrollment flow chart for KeY Treat.

A total of 1063 Kentuckians screened for the study, and 990 were at least 18 years of age and Perry County residents (Figure 1). Those eligible for the study were at least 18 years of age, a resident of the target county, and were HCV RNA‐positive. Pregnant people were excluded from study participation but were invited to re‐screen post‐pregnancy. Of the 990 eligible (>18 years, Perry County resident), 762 (77%) attended the clinic for the HCV RNA (see Havens et al.,^22^ for a description of screened individuals). About half (*N* = 380) of those screened were viremic and enrolled in the study. Two participants left after providing consent and did not return to complete study‐related activities, leaving a final sample size of 378 participants for the current analysis. Approval for the study protocol was provided by the Institutional Review Board (IRB) at the University of Kentucky. For the baseline visit, $50 in remuneration was provided to enrolled participants in addition to the $25 they received for screening.

## MEASURES AND VARIABLES

Three sources of data were utilized for this analysis—the eligibility screener, baseline survey, and laboratory measures. As part of the screening process, participants were asked “Have you ever been told you have hepatitis C”? The baseline survey was completed among those enrolled in KeY Treat and ascertained self‐reported demographic and drug use characteristics including age, race/ethnicity, gender, education, transportation, and health insurance. To assess lifetime and current drug use, injection drug use and recent (past 30‐day) use of prescription opioids, heroin, methamphetamine, cocaine, buprenorphine, marijuana and benzodiazepines to get high, the ASI Lite was utilized.^23^ Participants were also queried about lifetime and recent use of alcohol. To determine whether participants met the DSM‐IV criteria for major depressive disorder (MDD), generalized anxiety disorder (GAD) and post‐traumatic stress disorder (PTSD), the Mini‐International Neuropsychiatric Interview (M.I.N.I.) was administered.^24^ The baseline survey also queried self‐reported enrollment in MOUD (buprenorphine or methadone). To minimize respondent bias to sensitive questions around substance use, participants were regularly reminded via prompts in the survey that study participation did not require abstinence and that all responses were confidential and protected by a Certificate of Confidentiality issued by the National Institutes of Health.

## ON‐SITE LABORATORY MEASURES

Screening participants provided a capillary blood sample for the antibody tests for HCV (OraQuick Rapid Antibody Test, OraSure Technologies) and HIV (INSTI HIV‐1/HIV‐2 Antibody Test (bioLytical Laboratories) as well as the Xpert HCV VL Fingerstick assay (Cepheid). HCV RNA results from the Xpert VL Fingerstick assay were available in about 1 h. Because the VL Fingerstick assay was not FDA‐approved for use in the United States during study enrollment, the research team was granted a research exemption by Cepheid. The exemption required that enrolled participants receive confirmatory RNA peripheral blood testing. Thus, venipuncture was completed by the study phlebotomist. Samples were submitted to LabCorp for processing with results typically available within 48 h.

## OFF‐SITE LABORATORY MEASURES

Venous samples collected by the study phlebotomist were sent to LabCorp for processing and testing for HCV RNA, genotype, FibroSURE, CBC, confirmatory HIV, hepatitis A virus (HAV), and hepatitis B virus (HBV). Samples were labeled using numeric participant IDs. HBV and HAV infection and vaccine eligibility were determined using results from the LabCorp tests. If HAV total antibody and IgM tests were both negative, participants were offered the two‐series HAV vaccine (VATQA, Merck & Co., Inc.). For HBV, results from the hepatitis B surface antigen screen were utilized to determine whether participants had an active HBV infection, and if negative, the HBV surface antibody results were examined. If both were negative, the participant was offered the HBV three‐series vaccine (RECOMBIVAX HB, Merck & Co., Inc).

All laboratory results, whether obtained onsite or offsite, were entered into a secure Research Electronic Data Capture (REDCap)^25^, ^26^ database by the study phlebotomist. After the initial results were entered, their validity was verified by a second party, most often the study nurse or medical assistant. Paper charts were also maintained for each participant (identified only by participant ID) and printed laboratory results were stored within each chart.

## DATA ANALYSIS

For the current analysis, baseline sociodemographic, drug use, and clinical characteristics were examined. Two groups were compared—people who recently injected drugs (PWID) in the past 30 days and those who were not recently injecting drugs (non‐PWID). Contingency table and the chi‐square statistic were employed to examine differences in categorical variables across PWID/non‐PWID. The sample size for each group and proportion are presented for each variable by injection status. The Wilcoxon rank‐sum test was used to compare median values across the two injection groups. The positive percent agreement (PPA) was also calculated to compare the effectiveness of the Cepheid HCV VL Fingerstick assay versus the LabCorp assay (Cobas® AmpliPrep/Cobas® TaqMan® HCV Test v 2.0) in detecting HCV viral RNA. PPA was also calculated to compare self‐reported HCV status to antibody‐confirmed HCV status.

## RESULTS

### Demographics

Demographic characteristics of study participants are presented in Table 1. Age, race/ethnicity, education and marital status did not differ among people who reported recently injecting drugs and those who did not. However, a significantly smaller proportion of PWID were women (27.4%) compared to non‐PWID (46.0%; *p* = 0.016). Medicaid coverage was high among those enrolled in KeY Treat (93.6%), and while a slightly greater proportion of PWID were enrolled than non‐PWID (94.6% vs. 93.1%), the differences in proportions were not statistically significant. Access to a car was low across both groups (34.1%), but was significantly lower among PWID, where less than one in five participants had access to an automobile (17.8%), versus 42.7% of those without recent injection drug use (*p* < 0.001).

**TABLE 1:** Baseline sociodemographic and drug use characteristics for participants with recent (PWID) and without recent (non‐PWID) injection drug use.

	Overall *N* = 378		PWID *n* = 130		Non‐PWID *n* = 248		
	*n*	%	*n*	%	*n*	%	*p*‐value
Age in years, median (IQR)	41.8 (35.4, 48.7)		41.6 (35.3, 48.3)		41.9 (35.4, 49.1)		0.536
Female	157	41.5	43	27.4	114	46.0	0.016
White	365	96.6	124	95.4	241	97.2	0.364
Married	78	20.6	27	20.8	51	20.6	0.963
Education in years, median (IQR)	12 (10, 12)		12 (9.75, 12)		12 (10, 12)		0.323
Access to car	129	34.1	23	17.8	106	42.7	<0.001
Health insurance							
Medicaid/Medicare	354	93.6	123	94.6	231	93.1	0.810
Private insurance	9	2.4	3	2.3	6	4.2	
Uninsured	15	4.0	4	3.1	11	4.4	
Lifetime substance use							
Alcohol	337	89.1	113	86.9	224	90.3	0.313
Heroin	188	49.7	98	75.4	90	36.3	<0.001
Prescription opioids	337	89.1	120	92.3	217	87.5	0.153
Illicit buprenorphine	228	60.2	88	67.7	140	56.4	0.034
Methamphetamine	276	73.0	118	90.7	158	63.7	<0.001
Benzodiazepines	258	68.2	93	71.5	165	66.5	0.321
Cocaine	298	78.8	109	83.8	189	76.2	0.084
Marijuana	344	91.0	120	92.3	224	90.3	0.522
Lifetime injection (any substance)	344	91.0	130	100	214	86.3	<0.001
Methamphetamine	229	60.6	112	86.1	117	47.2	<0.001
Heroin	151	40.0	86	66.1	65	26.2	<0.001
Cocaine	225	59.5	90	69.2	135	54.4	0.005
Past 30‐day substance use							
Alcohol	71	18.9	30	23.1	41	16.5	0.122
Heroin	52	13.7	46	35.4	6	2.4	<0.001
Prescription opioids	16	4.2	8	6.1	8	3.2	0.179
Illicit buprenorphine	119	31.3	54	41.5	65	26.1	0.002
Methamphetamine	144	37.9	109	83.8	35	14.1	<0.001
Benzodiazepines	58	15.3	27	20.8	31	12.5	0.034
Cocaine	17	4.5	14	10.8	3	1.2	<0.001
Marijuana	180	47.4	87	66.9	93	37.5	<0.001
Past 30‐day injection							
Methamphetamine	102	26.8	102	78.5	–	–	
Heroin	45	11.8	45	34.6	–	–	
Cocaine	16	4.2	16	12.3	–	–	

### Drug use

As seen in Table 1, a third of those enrolled in KeY Treat (34.1%) injected drugs in the 30 days prior to the baseline. The most commonly injected drugs included methamphetamine (78.5%), followed by heroin (34.6%) and cocaine (12.3%). The vast majority of participants reported a lifetime history of injecting (91.0%). Three out of every five participants indicated lifetime methamphetamine and cocaine injection and those recent PWID were significantly more likely to have a lifetime history of methamphetamine, heroin and/or cocaine injection.

Lifetime and past 30‐day substance use is also presented in Table 1. Although substance use history was not part of the eligibility requirements,100% of participants reported lifetime use of any substance. Lifetime non‐medical use of prescription opioids (89.1%) and marijuana (91.0%) was most commonly mentioned, followed closely by cocaine (78.8%) and methamphetamine (73.0%). Also noted were the lower rates of recent prescription opioid use (4.2%) versus lifetime use (89.1%), which demonstrates the shift in drug use from prescription opioids to methamphetamine in recent years. The most commonly used drug in the past 30 days among all participants was marijuana (47.4%). However, about a third of participants also endorsed past 30‐day use of methamphetamine (37.9%); use was significantly higher among PWID (83.8%) versus non‐PWID (14.1%, *p* < 0.001).

Lifetime and recent use of illicit buprenorphine (distinguished in the interview from licitly obtained buprenorphine from a licensed provider) was frequently reported by KeY Treat participants (Table 1). More than half (60.2%) of KeY Treat enrollees indicated lifetime use of illicit buprenorphine with a third also reporting past 30‐day use (31.3%). Lifetime and recent illicit buprenorphine use was reported at significantly higher rates among PWID (41.5%) than non‐PWID (26.1%, *p* = 0.002).

### Clinical characteristics

Results from the FibroSURE (Table 2) indicated that most KeY Treat participants had minimal liver damage (94.4% were F2 or less) and that PWID were actually less likely to be cirrhotic (F3 or F4 ‐ 2.3% vs. 7.3% for non‐PWID, *p* = 0.046) than non‐PWID. HIV and HBV infections were rare among KeY Treat participants. Only two patients presented with a laboratory‐confirmed HIV infection (both among PWID, *p* = 0.05 compared with 0 infections among non‐PWID; both were already undergoing treatment for their HIV infection) and six with HBV. The proportion of HBV‐positive individuals did not differ among PWID and non‐PWID.

**TABLE 2:** Baseline clinical characteristics among people who inject drugs with a recent history (PWID) and those without a recent history of injecting drugs (non‐PWID).

	Total sample *N* = 378		Recent PWID *n* = 130		Non‐PWID *n* = 248		
	*n*	%	*n*	%	*n*	%	*p*‐value
Quantitative HCV RNA, median (IQR)	1,573,000 (901,250, 2,662,500)		1,522,500 (759,000, 2,683,500)		1,587,000 (930,000, 2,671,500)		0.595
Genotype (*n* = 351)*							
1a	223	63.2	67	58.8	155	65.4	0.581
1b	5	1.4	1	0.9	4	1.7	
1a/3	1	0.3	1	0.9	0	0	
2a/2c	1	0.3	0	0	1	0.4	
2b	36	10.3	12	10.5	24	10.1	
3	82	23.4	32	28.1	50	21.1	
4	4	1.1	1	0.9	3	1.3	
FibroSURE staging							
F0–F2	354	94.4	126	97.7	228	92.7	0.046
F3–F4/F4	21	5.6	3	2.3	18	7.3	
HIV‐positive	2	0.5	2	1.5	0	0	0.050
HBV‐positive	6	1.6	3	2.3	3	1.2	0.417
Albumin, median (IQR)	4.4 (4.2, 4.7)		4.4 (4.2, 4.6)		4.4 (4.2, 4.7)		0.500
Bilirubin, median (IQR)	0.4 (0.3, 0.5)		0.4 (0.2, 0.5)		0.3 (0.3, 0.5)		0.530
PT	10.7 (10.4, 11.1)		10.7 (10.3, 11.1)		10.8 (10.4, 11.1)		0.732
INR	1 (1, 1.1)		1 (1, 1.1)		1 (1, 1.1)		0.533
AST U/L	36.5 (27, 58.5)		39 (29, 55.5)		35 (26, 61)		0.688
ALT U/L	43 (27.25, 71.75)		43 (29, 71.5)		42 (26, 73)		0.617
HAV vaccine eligible	126	35.3	35	29.4	91	38.4	0.094
HBV vaccine eligible	112	31.7	26	22.0	86	36.6	0.006
HBsAg‐positive	6	1.6	2	1.4	4	1.7	0.819
Enrolled in MOUD Tx	175	47.0	41	31.8	133	55.0	<0.001
Buprenorphine	134	36.0	34	26.4	99	40.9	0.005
Methadone	42	11.3	7	5.4	35	14.5	0.009
M.I.N.I. Mental Health							
MDD	104	27.5	60	46.1	44	17.7	<0.001
GAD	99	26.1	52	40.0	47	18.9	<0.001
PTSD	50	13.2	22	16.9	28	11.3	0.125

* Quantitative RNA level was insufficient to reflex to determine genotype for all participants.

Approximately one‐third of participants were eligible for HAV (35.3%) and HBV (31.7%) vaccines. A significantly smaller proportion of PWID were eligible for the HBV vaccine (22% vs. 36.6% for non‐PWID, *p* = 0.006), indicating more PWID were either previously vaccinated against the virus or were immune via a prior infection. While a greater number of non‐PWID were eligible for the HAV vaccine (38.4% vs. 29.4% for PWID), the difference in proportions of those eligible was not statistically significant.

Current enrollment in MOUD treatment was common among KeY Treat participants (47%); almost half of those consenting to participation in the trial indicated they were receiving MOUD at the baseline study visit. While this proportion was significantly lower among active PWID, it was not zero. In fact, almost one in three PWID were enrolled in MOUD (31.8%) compared to 55% of non‐PWID (*p* < 0.001). Buprenorphine was the most commonly utilized medication for the treatment of opioid use disorder, as 76.6% of those receiving MOUD were prescribed buprenorphine.

One‐quarter of enrolled KeY Treat participants met diagnostic criteria for major depressive disorder (MDD), and a far greater proportion of PWID (46.1%) met the DSM‐IV criteria for MDD than non‐PWID (17.7%, *p* < 0.001). Similar trends were observed for generalized anxiety disorder (GAD), where 40% of PWID and only 18.9% of non‐PWID met diagnostic criteria for GAD (*p* < 0.001). Post‐traumatic stress disorder was less prevalent overall and there were no differences in the proportion screening positive for PTSD among PWID and non‐PWID.

**FIGURE 2:**
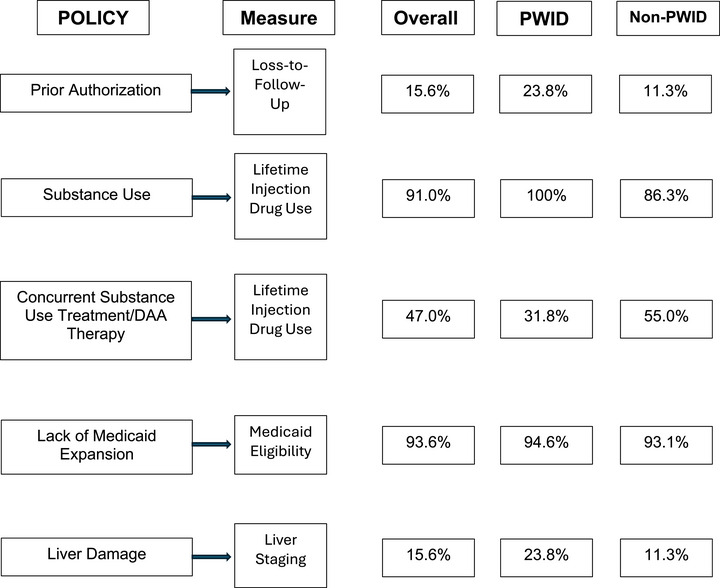
KeY Treat participant characteristics in the context of Medicaid policies.

Results salient to state Medicaid policies (prior authorization, substance use/substance use treatment, Medicaid expansion, and liver staging) for accessing HCV treatment are presented in Figure 2. Prior authorization often requires multiple visits before DAA initiation; in KeY Treat, more than 15% of participants were lost‐to‐follow‐up after the first visit. States may require abstinence and/or concurrent enrollment in substance use treatment as part of HCV care. In KeY Treat, the overwhelming majority of participants have a history of drug injection and less than half were enrolled in MOUD at baseline. Most KeY Treat participants were eligible for Medicaid (93.6%), which would impact DAA access in non‐Medicaid expansion states. Finally, liver staging indicative of lesser liver damage was highly prevalent in KeY Treat. More than 90% of participants had little to no liver damage, a finding that may have precluded them from receiving DAA treatment in years past, including the first few years of KeY Treat enrollment.

Finally, there was 100% agreement between participant's prior knowledge of their hepatitis C status and testing anti‐HCV positive. Additionally, the effectiveness of the GeneXpert VL Fingerstick assay was assessed versus the LabCorp Cobas® AmpliPrep/Cobas® TaqMan® HCV Test v 2.0. The positive percent agreement (PPA) in detecting viral RNA between the Xpert point‐of‐care fingerstick assay and LabCorp Cobas assay was 100%.

## DISCUSSION

Results from this study suggest that, given the current Medicaid landscape across the United States, access to curative HCV treatments may still be limited for some individuals. All KeY Treat participants had a history of substance use and more than a third reported injecting drugs in the 30 days prior to enrollment. Almost half of KeY Treat participants were using drugs at baseline and a third were actively injecting in the 30 days leading up to study enrollment. Virtually all enrolled in the trial had a lifetime history of drug injection. While most substance use restrictions for accessing HCV care in Medicaid programs have been removed, six states still have some provision whereby substance use needs to be addressed prior to HCV treatment initiation.^12^ There is also significant stigma around accessing HCV treatment for people who are using drugs or with a history of substance use^27^, ^28^ that is further exacerbated by the reticence of clinicians to treat HCV tied to concerns about reinfection, especially among active PWID.^10^, ^29^ Importantly, a recent meta‐analysis of 36 studies as well as the HERO study demonstrated that HCV reinfection rates are low among PWID achieving initial cure with DAAs, especially among those enrolled in MOUD treatment.^30^, ^31^ However, one‐quarter of US states have retreatment restrictions,^12^ which essentially means that if a patient is reinfected, subsequent HCV treatment may not be covered by Medicaid. As reinfection rates are low, this restriction appears biased against PWID and risks increasing stigma around (and decreasing interest in) HCV treatment seeking in this marginalized population.^32^ Further, treating PWID is the key to elimination of the hepatitis C virus ^4^, ^33^, ^34^, ^35^ and removing retreatment restrictions would go a long way in encouraging clinicians to treat this vitally important population of people living with HCV.^36^, ^37^, ^38^, ^39^, ^40^, ^41^

While many restrictions in state Medicaid programs have been removed as of 2025,^12^ accessing Medicaid benefits is still problematic among those who reside in non‐expansion states. As seen in KeY Treat, the overwhelming majority (93.6%) of those seeking treatment for HCV were enrolled in Medicaid. While Kentucky saw a dramatic increase in Medicaid enrollment after expansion, even among people who use drugs,^15^ Medicaid expansion has not been adopted nationwide, and an estimated 1.6 million Americans lack coverage.^42^ So even in states where HCV treatment restrictions have been reduced or removed, the inability to access Medicaid benefits among PWID in non‐expansion states is likely hampering HCV treatment access,^14^ as evidenced by a significantly lower rate of people treated with DAAs in non‐expansion states.^41^

In the absence of universal insurance coverage, efforts to cover HCV treatment costs, regardless of insurance status, should be made. Numerous studies have found that it is cost‐effective to treat HCV among PWID in a variety of settings,^43^, ^44^, ^45^, ^46^, ^47^ especially when paired with harm reduction services.^48^ Australia and Georgia have both undertaken country‐wide elimination efforts whereby DAAs are available to all RNA‐positive residents.^46^, ^49^, ^50^, ^51^ Treatment is not reliant on insurance coverage in these countries. For example, the Australian government negotiated directly with pharmaceutical companies for an unlimited supply of DAA for 5 years (2016–2021) at a cost of 1.2 billion Australian dollars seeing the potential for large cost savings by preventing more costly complications of untreated HCV.^46^ A critical driver of DAA cost is a country's ability to negotiate directly with pharmaceutical companies for affordable pricing; thus, the United States may benefit from policy allowing for more directed negotiations.^52^ In addition, both countries utilize traditional medical providers as well as non‐traditional venues, such as harm reduction service providers, emergency departments, and substance use disorder treatment service providers, to identify and/or treat PWID infected with HCV.^50^, ^53^, ^54^, ^55^, ^56^ This is certainly a model that would bypass the differing state‐level Medicaid requirements as well as lack of Medicaid expansion in some states that is hampering HCV treatment efforts. Many of these key characteristics have been adopted in the Cure Hepatitis Act of 2025,^38^ which includes blueprints for a national plan whereby Medicaid restrictions are removed and a subscription plan is implemented for purchasing DAAs. There are also provisions to have drug on site in traditional and non‐traditional (harm reduction programs, MOUD clinics) settings to meet people living with HCV where they are at.

The PPA comparing prior knowledge of HCV status and antibody testing was 100%, suggesting that patient report of positive HCV status may negate the need to conduct antibody testing. This further reduces the diagnostic burden and allows clinicians to move directly to RNA testing among those reporting prior knowledge of HCV infection. The positive percent agreement between Cepheid's fingerstick point‐of‐care test and the Cobas assay was 100%, demonstrating the efficacy of the Xpert device in identifying treatment‐eligible individuals. Same‐day test and treat models, which are likely the most effective way to engage PWID in curative treatment,^57^, ^58^ are now possible in the United States with the recent FDA approval of this CLIA‐waived, point‐of‐care assay.^59^ However, the 18 states with prior authorization requirements will not be able to fully capitalize on same‐day test and treat models in care,^60^ as physicians report that prior authorizations often create delays in care.^40^ Even though KeY Treat employed a same‐day test and treat model of care, negating the need for prior authorization and multiple visits before initiating DAA treatment, we still saw significant loss‐to‐follow‐up in the first two weeks of treatment. This drop off from the prior authorization requirement is likely higher in clinical practice without the extensive retention protocols used in KeY Treat. Getting the medications into the hands of those at greatest risk for transmission is a vital piece of the elimination puzzle,^58^ and the prior authorization requirements severely hamper those efforts. While most of the KeY Treat participants would have likely been eligible for treatment in states with prior authorization requirements, this extra step negates clinicians’ ability to employ a test and treat model. Prior authorizations also promote bias among providers, as a recent study found that requiring clinicians to obtain prior authorization was negatively associated with DAA prescribing.^10^

Lastly, the absence of a rapid, point‐of‐care test for hepatitis B virus (HBV) plays a part in upholding barriers to accessing DAA treatment given the potential for HBV reactivation during and after DAA treatment.^61^ Prior authorization for DAA therapy in Kentucky, for example, requires testing for evidence of hepatitis B virus infections.^62^ HBV reactivation is also a concern for test and treat models in that DAA initiation begins without knowledge of current or prior HBV infection. However, a recent study of DAA recipients with resolved infections,^63^ as well as a meta‐analysis^64^ and systematic review,^65^ all suggest low rates of HBV reactivation. Data from KeY Treat suggest that the overall prevalence of HBV is quite low (1.6%) in this population of rural Appalachians and that the overwhelming majority of participants would benefit from same‐day DAA initiation without the need for prior authorization.

### Limitations

This study is not without limitations. First, the study sample represents one county in Appalachian Kentucky, and therefore, results may not be generalizable to other regions of the United States. However, what occurred in Appalachia in the past two decades was not unique, and the negative impacts of the opioid epidemic, including the opioid/HCV syndemic, were widespread throughout the United States. In addition, restrictions around accessing DAAs among Medicaid recipients is an ever‐changing landscape and the applicability of some of the findings may change as the needs of those seeking care are addressed.

## CONCLUSION

Results from the current study demonstrate the need for a national plan that removes all state restrictions for accessing HCV treatment, even in states without Medicaid expansion, such as what is proposed in the Cure Hepatitis Act of 2025.^38^ As a national HCV elimination plan takes shape in the United States, it will be necessary to remove barriers to care through national policy changes, including elimination of preauthorization requirements, removal of retreatment restrictions, and negotiation of DAA drug prices in order to effectively reach those in need of care and eliminate HCV in the United States.

## CONFLICT OF INTEREST STATEMENT

Jennifer Havens has served as a scientific consultant for Gilead Sciences, Inc., and Cepheid.
